# The role of hypoxia-inducible factor-1 alpha in multidrug-resistant breast cancer

**DOI:** 10.3389/fonc.2022.964934

**Published:** 2022-08-08

**Authors:** Liyun Yong, Shasha Tang, Haixin Yu, Hongyi Zhang, Yi Zhang, Yuan Wan, Fengfeng Cai

**Affiliations:** ^1^ Department of Breast Surgery, Yangpu Hospital, School of Medicine, Tongji University, Shanghai, China; ^2^ Department of Orthopedic Surgery, Yangpu Hospital, School of Medicine, Tongji University, Shanghai, China; ^3^ The Pq Laboratory of BiomeDx/Rx, Department of Biomedical Engineering, Binghamton University—SUNY, Binghamton, NY, United States

**Keywords:** breast cancer, drug, resistance, inhibition, hypoxia inducible factor-1 Alpha (HIF-1α), targeted drugs

## Abstract

Breast cancer is the most common cancer in women worldwide with increasing incidence. Significant therapeutics advances in the field of breast cancer have resulted in a growing number of treatment options, whereas *de novo* or acquired resistance is still a persistent clinical challenge. Drug resistance involves a variety of mechanisms, and hypoxia is one of the many causes. Hypoxia-inducible Factor-1 Alpha (HIF-1α) is a key transcription factor which can regulate the response of cells to hypoxia. HIF-1α can trigger anaerobic glycolysis of tumor cells, induce angiogenesis, promote the proliferation, invasion, and migration of tumor cells, and lead to multidrug resistance. This review mainly discusses the role of HIF-1α in the drug-resistant breast cancer and highlighted the potential of HIF-1α -targeted therapy.

## Introduction

Breast cancer is the most common malignancy in women and the second leading cause of female cancer-related death after lung cancer (1). Its therapy methods mainly include surgery, endocrine therapy, chemotherapy, radiotherapy and targeted therapy based on the classification of tumors, among which drug therapy occupies an important part of the treatment of breast cancer. In the early 1990s, breast cancer mortality had declined due to its reduction in the risk, improvements in treatment and widespread use of early screening (2). However, the emergence of drug resistance during treatment in recent years has brought severe challenges for the survival of breast cancer patients (3). Resistance to anticancer drug therapy is caused by a variety of factors, which include tumor burden and growth kinetics; tumor heterogeneity; physical barriers; undruggable cancer drivers; the many consequences of applying therapeutic pressures; the immune system and the microenvironment with hypoxia (4, 5). Hypoxia in the tumor microenvironment refers to a condition where the pressure of oxygen is lower than 5–10 mm Hg (6). Hypoxia is caused by an imbalance between oxygen consumption and oxygen supply due to rapid growth of tumor (7). As a hallmark of the tumor microenvironment, hypoxia occurs in a variety of tumors. It is well known that tumor hypoxia has a negative impact on treatment outcomes and prognosis. Hypoxia inhibits tumor cell proliferation, induces cell cycle arrest, and ultimately develops drug resistance because anticancer drugs preferentially target cells that are rapidly proliferating (8).

Hypoxia-inducible factor-1 (HIF-1) is a transcription factor that responds to hypoxia and is involved in several aspects of tumor progression, including metastasis, angiogenesis, drug resistance, and immune evasion (9). The constitutive HIF-1β/ARNT subunit and the highly oxygen-sensitive HIF-1α subunit constitute the HIF1 protein, while intracellular HIF-1α levels determine the activity of HIF-1 (10). After HIF-1α was initially discovered by identifying a hypoxia response element (HRE) in the 1990s, scholars have proven that it is a key regulator responsible for the induction of genes that facilitate adaptation and survival under low oxygen conditions (11). Overexpression of hypoxia-inducible factor-1 alpha (HIF-1α) is associated with drug resistance, poor prognosis, and a higher risk of metastasis in breast cancer patients (12). Currently, numerous small molecule inhibitors are under development, some of which are considered in clinical trials. For instance, in a phase II trial of echinomycin, a HIF-1α transcription inhibitor for metastatic non-small cell lung cancer, the response rate of patients treated with echinomycin was 5%, and the median survival was 24.3 weeks (13). Although the treatment did not satisfy the predefined expectations during the time, it revealed the feasibility of using HIF-1α as a potential target for cancer treatment. As research and development of drugs targeting HIF-1α are primarily based on its mechanism of action, exploring this aspect of breast cancer drug resistance is of great significance to the development of related drugs. It can provide a reference value for clinical combination therapy. Therefore, this review aims to investigate the role of HIF-1α in treating breast cancer drug resistance, emphasizing its potential as a therapeutic target, and forecast its inhibitors and clinical application prospects.

## Structure of HIF-1α

**Figure 1:**
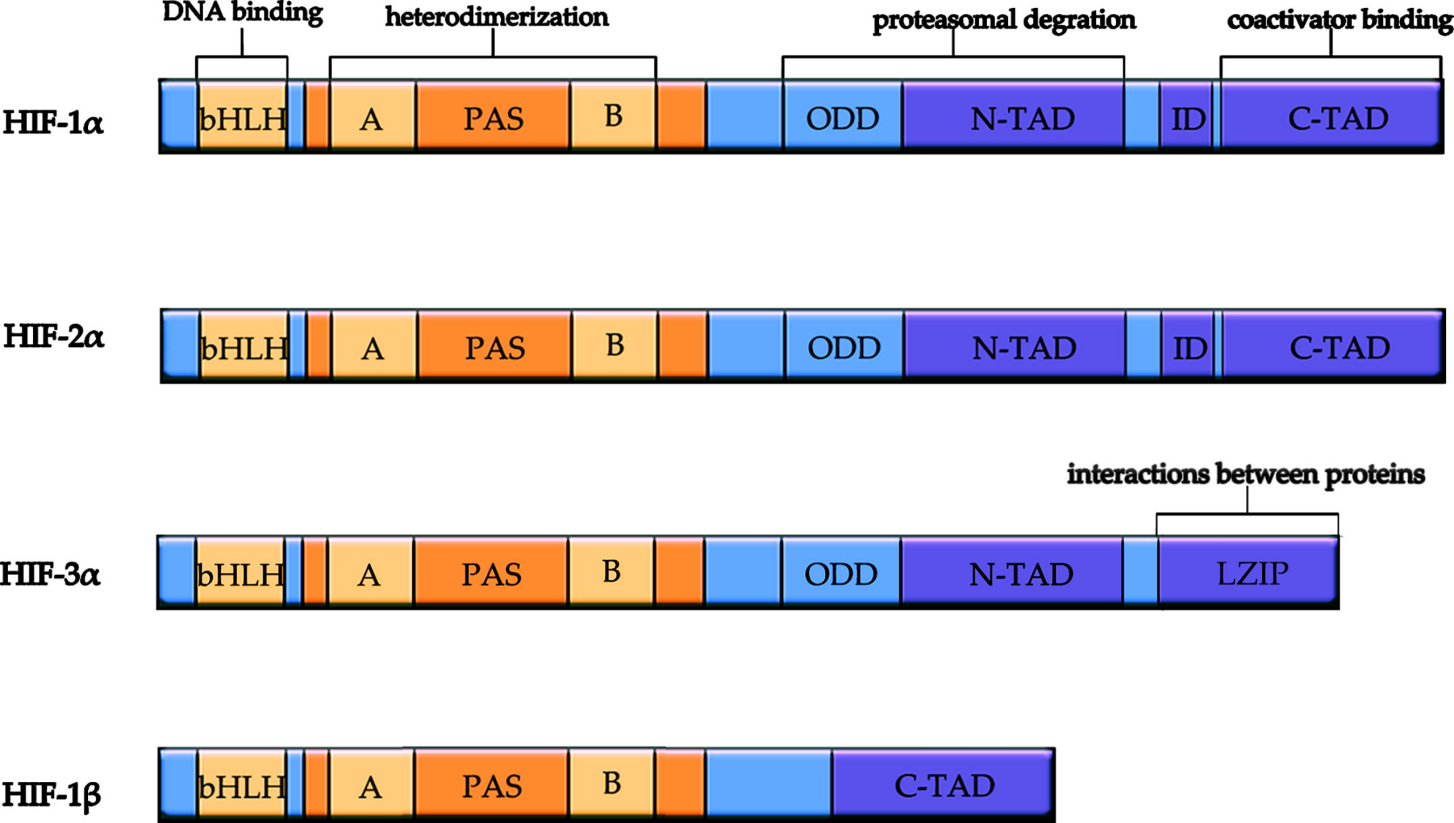
Schematic diagram of structure and function of HIF subunits. All HIF isoforms have a bHLH motif and two PAS domains responsible for heterodimerization. HIF-3α replaces C-terminal trans-activation domain with C-terminal Leucine zipper (LZIP) domain responsible for interactions between proteins. HIF-1β does not contain ODD domain for proteasomal degradation, N-TAD, and ID. bHLH, basic helix-loop-helix domain; PAS, Per/ARNT/Sim domain; ODD, oxygen-dependent degradation domain; ID, inhibitory domain. N-TAD, N-terminal transactivation domain; C-TAD, C-terminal transactivation domain.

Hypoxia is involved in many pathological and physiological processes of the human body and acts as an important regulator. HIFs are an integral component of tumor adaptation in the hypoxic tumor microenvironment (14). So far, three types of HIFs have been identified in mammals. HIFs are heterodimeric proteins composed of an O_2_-sensitive α subunit (HIF-1α, HIF-2α, and HIF-3α) and an O_2_-insensitive β subunit (HIF-1β) and play a key role in the regulation of many genes transcribed in hypoxic conditions (**Figure 1**) (15). All three HIF-α genes are regulated by oxygen and bind to HIF-1β, but only HIF-1α and HIF-2α have been extensively studied (16). Although HIF-1α and HIF-2α share similar amino acid sequences and bind to the same HRE, they differ in several aspects (17).. First, HIF-1α is widely expressed, while HIF-2α is relatively tissue-specific (18). Second, some studies showed that the oxygen dependence of HIF-1α and HIF-2α significantly differed as HIF-1α was more active and lasted for a shorter time under severe hypoxia, whereas HIF-2α was more active and lasted longer under moderate hypoxia (19–21).

**Figure 2:**
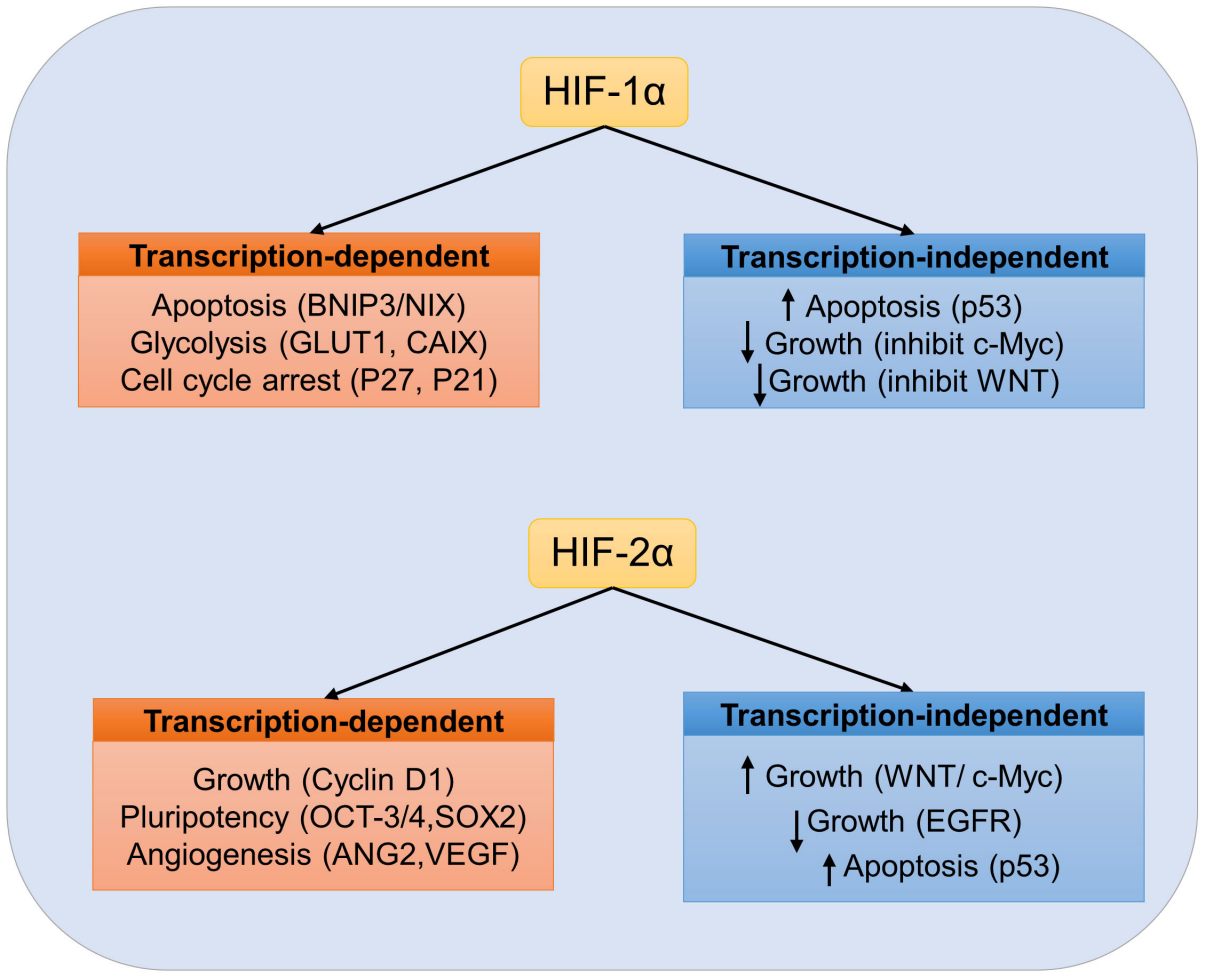
Specific role of HIF in tumor cells. Transcription-dependent and independent targets of HIF-1α or HIF-2α are listed. No overlapping HIF targets are listed. Depending on the cellular environment, activation of HIF transcription factors may have pro-tumor or anti-tumor effects.

HIF-1α and HIF-2α show non-overlapping antagonistic roles due to their unique regulators, different expression patterns, and gene targets (22). HIF-1α is ubiquitously expressed in hypoxic tissues, whereas HIF-2α is mainly expressed in a certain cell types, including vascular endothelial cells (ECs) and macrophages (23). The HIF transcription factors show disparate spatiotemporal regulation. For example, HIF-1α can be activated under acute and severe hypoxia (1-2% O_2_), whereas HIF-2α is gradually accumulated under moderate hypoxia (5% O_2_) (24). Moreover, genes which regulate cell death or anaerobic glycolysis appear to be predominantly controlled by HIF-1α, but genes which regulate erythropoietin synthesis (EPO) and tumor stemness or pluripotency are primarily regulated by HIF-2α (10). Furthermore, as to typical HRE mediated transcription, HIF subtypes also differentially regulate signaling pathways by interacting with proteins that do not contain PAS domains, such as β-catenin, p53, Notch intracellular domains, and c-myc proto-oncogene (23, 25, 26). Emerging data suggests that the HIF-α subtype is specific in multiple solid tumor types *e.g.*, glioblastoma, kidney carcinoma, and neuroblastoma, and HIF-α subtype may promote tumor progression (**Figure 2**) (23).

At present, there are few studies on HIF-3α, which may be related to the complex function due to a large number of different variants (27). The C-terminal leucine zipper (LZIP) domain responsible for the interaction between proteins was found to replace C-terminal trans-activated domain in a HIF-3α variant (28). It is generally believed that the HIF-3α gene is expressed as a selective splicing isomer, which can activate or inhibit HIF target gene (27).

HIF-1 contains HIF-1α subunit with 826 amino acids and the HIF-1β subunit with 782 amino acids. Both subunits belong to the basic helix-loop-helix/Per-ARNT-Sim (bHLH-PAS) family of transcription factors (29). N-TAD (N-terminal TAD) and C-TAD (C-terminal TAD), located in HIF-1α, are two transactivation domains (TADs) with rich acidic and hydrophobic amino acids (30). C-TAD is mainly responsible for regulating HIF-α transcription by interacting with the transcriptional co-activator protein CREB binding protein/P300 under hypoxia, whereas N-TAD is mainly a regulator for its stabilization (31, 32). The regions between the two TAD sequences are inhibitory domains (ID; Amino acids 576-785), which inhibit the transcriptional activation of TAD (33). HIF-1α contains the oxygen-dependent degradation (ODD) domain in upstream of the N-TAD region responsible for its degradation by the ubiquitin-proteasome pathway (34). HIF-1β (also known as aryl hydrocarbon receptor nuclear translocator ARNT) is constitutively expressed in all cell types and is not regulated by oxygen levels (35). HIF-1β subunit lacks ODD and N-TAD domains and contains only C-TAD, and its structural differences are reflected in its function (30).

## Induction of HIF-1α in breast cancer

HIF-1α stabilization was reported in tumors of varying origins, and functional analyses led to the perception of HIF-1α as an oncoprotein (36). HIF-1α is located in the cytoplasm and is easily degradable under normoxia conditions with a half-life of less than 5 min. However, many studies have found that HIF-1α enhances stability in the presence of hypoxia and maintains a set of mechanisms for stability and activation in the presence of normoxia. Hence, the mechanisms of HIF-1α stabilization and transcriptional activation under normal and hypoxic conditions are discussed based on: (1) classical oxygen-dependent pathways and (2) oxygen-independent pathways.

### Classical oxygen-dependent pathways

Under normal physiological conditions, HIF-1α is degraded in the body and cannot exert its biological effects. Hypoxia-inducer -α (HIF-α) protein inactivation is mainly regulated by FIH-1 and PHD through interactions with their specific N-TAD and C-TAD domains (37). FIH-1 is an oxygen-dependent enzyme that hydroxylates aspartic acid residues at position 803 (Asn803) in the transactivation domain of the HIF-1α C-terminal. The transcriptional activation function of HIF-1α was inhibited by blocking the binding of HIF-1α with CBP (CREB-binding protein)/P300 (38). Prolyl hydroxylase (PHDs) is also an oxygen-dependent enzyme that hydroxylates the key residue Pro564 and Pro402 of HIF-1α, located in the oxygen-dependent degradation domain (39). Subsequently, the E3 ubiquitin ligase Von Hippel Lindau protein (pVHL) binds to the ODD domain of HIF-1α subunit, recruiting a variety of ubiquitin proteins to form the ubiquitin ligase complex, leading to ubiquitination of the HIF-1α subunit (40). Finally, HIF-1α is degraded by the ubiquitin-linked protease complex pathway. The expression of PHDs varies from tissue to tissue, and the affinity for different HIF proteins varies, which may lead to the diversity of hypoxic responses. In addition to hydroxylation of Pro564, Pro402, and Asn803, lysine (Lys532) in the oxygen-dependent degradation domain is blocked by acetyltransferase arrest-defective 1 (ARD1) to promote tumor pVHL binding, leading to HIF-1α instability (41).

**Figure 3:**
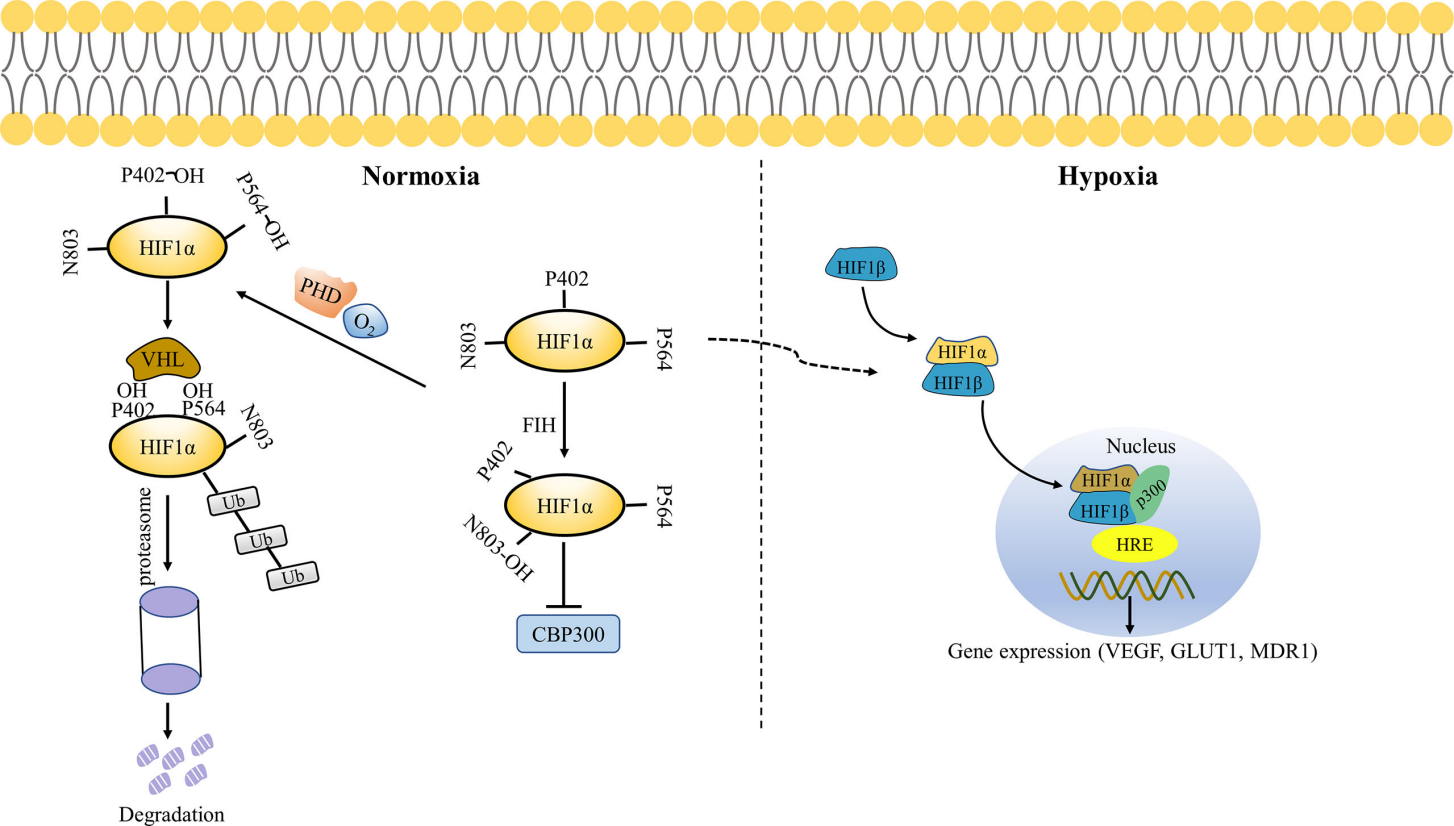
Oxygen-dependent regulation of HIF-1α. Under normoxic conditions, FIH hydroxylates HIF-1α Asn803 residues and blocks the binding of HIF-1α to CBP/P300, thereby inhibiting its transcriptional activation. PHD hydroxylates the key residues Pro564 and Pro402 of HIF-1α, resulting in pVHL binding to HIF-1α and ubiquitination of HIF-1α, which is ultimately degraded by the proteasome. Under hypoxia conditions, FIH and PHD are inactivated. HIF-1α and HIF-1β translocate to the nucleus, thus binding to p300 and hypoxia response elements (HRE) in the nucleus to activate gene transcription.

Under hypoxic conditions, FIH-1and PHDs activity is inhibited, resulting in decreased HIF-1α hydroxylation and repressed proteasomal degradation (42). HIF-1α stabilizes and dimerizes with HIF-1β present in the cytoplasm and nucleus of anoxic and normal cells to form HIF-1, which is then translocated to the nucleus (43). Heterodimer HIF-1 and co-activator CREB binding protein/P300 bind to hypoxia response element (HRE), which activates transcriptional activity of target genes such as VEGF, GLUT1, and MDR1 (**Figure 3**) (44). Therefore, HIF-1α does not degrade, leading to a rapid increase in intracellular protein levels (38).

### Oxygen-independent pathways

Most current studies focused on the relationship between cancer and HIF-1α in the context of hypoxia have limited insight because about 50% of advanced solid tumors lack hypoxic zones, and as a result, they remain able to activate HIF-1α (42). Therefore, exploring the stabilization and activation mechanism of HIF-1α in non-hypoxia conditions might help us to have a comprehensive understanding of its role in tumorigenesis, thus providing new targets for treatment.

Several pathways regulate HIF-1α stabilization and are thought to contribute to the intracellular accumulation of HIF-1α. For instance, it was reported that extracellular-signal-regulated kinase (ERK) was involved in the regulation of HIF-1α synthesis and transcriptional activation (45). In addition, ERK phosphorylates the co-activator CBP/P300 and increases the formation of HIF-1α/P300 complex, thereby stimulating its transcriptional activation (46). Some common genetic alterations in the oxygen-signaling pathway, such as loss of tumor suppressors p53, PTEN, and pVHL increase HIF-1α transcription, translation, or stability independently of O2 levels leading to tumor progression (47–49). A previous study reported that the loss of p53 enhances HIF-1α levels in human colon cancer, which may be explained by the role of p53 in promoting Mdm2-mediated ubiquitination and proteasomal degradation of the HIF-1α (50). Similarly, PTEN expression inhibited HIF-1α stabilization in glioblastoma-derived cell lines with evidence suggesting AKT regulation involvement, although AKT is indirectly associated with HIF-1α phosphorylation (51). As mentioned above, pVHL plays an important role in HIF-1α degradation. It was reported that HIF-1α stability was maintained, and HIF-1 was activated in VHL-deficient cells (52).Hsp90 inhibitors promoted effective ubiquitination and proteasome-mediated degradation of HIF-1α in RCC under normoxic and hypoxic conditions (53). Hsp90 directly binds to PAS domain of HIF-1α to induce conformational changes that enable HIF-1α to bind to HIF-1β, thereby initiating HIF-1α transactivation (54). Moreover, Hsp90 can stabilize HIF-1α by inhibiting its degradation.

## TME and HIF signaling and angiogenesis

Tumor microenvironment (TME) refers to the local biological environment of a solid tumor, consisting of both tumor cells, non-tumor cells, and extracellular matrix (ECM). In TME, there is a complex interaction and balance between tumor cells and non-tumor cells (55). Hypoxia, a hallmark of the TME, is caused by an imbalance between oxygen consumption and oxygen supply because of rapid tumor growth, which occurs in a variety of tumors including breast cancer (7). A key feature of the cellular response to hypoxia is the upregulation of multiple genes that promote angiogenesis/vascularization to increase oxygen delivery. This process is mediated by hypoxia-inducible factor (HIF-1α subunit) which can activate transcriptional responses under hypoxia (56, 57). Hypoxia-induced HIF-1α stabilization and accumulation can promote angiogenesis by increasing the expression of multiple pro-angiogenic genes. Vascular endothelial cell growth factor (VEGF) is one of its main target genes and is considered to be the main driver of angiogenesis. Particularly, VEGF can recruit endothelial cells to hypoxic and non-vascular areas and promote their proliferation (58). In addition to VEGF, HIF-1α regulates the expression of other angiogenic inducers (e.g., FGF, PDGF, and Ang-1/2) and angiogenic receptors (e.g., VEGFR, ANGPT receptor) (59–61). Meanwhile, HIF-2α plays an indispensable role in angiogenesis, which promotes vascular maturation (62).

HIF-1a not only mediates breast cancer angiogenesis but also leads to its metastasis, drug resistance, and poor prognosis. For example, HIF-1α signaling selectively supports breast cancer proliferation in the brain, which has been validated *in vivo* (63). In this study, nuclear HIF-1α staining was performed on breast cancer CTC-derived tumors growing in the brain and mammary gland, respectively. The results revealed that HIF-1α staining was approximately 11-fold increase in brain tumors in comparison with that in mammary tumors. Another study reported that mammary gland-specific deletion of Axl which is an HIF target can reduce HIF-1α levels in a HER2 + mouse model of breast cancer, thereby leading to a normalization of the blood vessels, a proinflammatory TME, and a reduction of lung metastases by inhibiting the hypoxia response of tumor cells (64). The *in vivo* data strongly suggests that HIF-1α plays a significant role in breast cancer metastasis.

In addition, some clinical randomized trials have demonstrated that HIF-1α can be used as a marker of poor prognosis and an independent predictor of drug resistance. For example, a clinical trial of 187 patients with T2-4 N0-1 breast cancer found that overall response to epirubicin and tamoxifen treatment decreased with increased tumor HIF-1α. The Kaplan-Meier curves showed that increased HIF-1α expression was associated with a significantly shorter disease-free survival (DFS) (65). Another clinical study enrolled 114 patients with T2-4 N0-1, estrogen receptor (ER) -positive breast cancer who were treated with letrozole. The response was assessed by measuring tumor size and detecting the presence of tumor cells in breast and axillary lymph nodes. The results found that 91 patients (81%) achieved disease response, 48 patients achieved complete clinical response (43%), and 22 patients did not achieve response (19%). Moreover, increased P44/42 MAPK and HIF-1α were found in patients without remission, suggesting that the increase in P44/42 MAPK and HIF-1α was a significant factor in treatment resistance in all leave-one-out iterations (63). A previous study also revealed that increased HIF-1α expression was associated with tamoxifen resistance. HIF-1α positivity was more common in contralateral breast cancer (CBC) during tamoxifen adjuvant therapy (N = 60) than in CBC without prior tamoxifen (N = 522) (32% (18/56) versus 17% (80/482) (64) These reports highlight the role of HIF-1α as a prognostic marker, but also demonstrate the positive association between HIF-1α overexpression and endocrine therapy resistance in breast cancer.

## HIF-1α contributes to drug resistance in breast cancer

Breast cancer is the most common malignancy in women and the second leading cause of female cancer-related death after lung cancer (1). Its therapy methods mainly include surgery, endocrine therapy, chemotherapy, radiotherapy, and targeted therapy based on the classification of tumors, among which drug therapy occupies an important part. However, drug resistance has become a major challenge in breast cancer treatment. Although the relationship between HIF-1α and drug resistance in breast cancer has been emphasized above, the mechanisms by which it induces resistance in chemotherapy, endocrine therapy, and targeted therapy remain to be clarified. This may be because HIF-1α is involved in various life activities in human cells. Studies suggested that HIF-1α may develop resistance to conventional therapies through a series of signaling pathways including drug effusion, tumor stem cell enrichment, autophagy and apoptosis (7, 66). Therefore, we will explore the mechanism of HIF-1α leading to breast cancer drug resistance from the above mentioned related signaling pathways.

### HIF-1α mediated overexpression of drug efflux proteins

A major cause of cancer MDR is the increased efflux of various ATP-dependent hydrophobic cytotoxic drugs, mediated by transmembrane transporters of ATP binding cassette (ABC) superfamily (67). ABC transporters are known as a complete family of membrane proteins, including many recognized drug transporters, such as the well-known multidrug resistance 1 protein (MDR1)/P-glycoprotein encoded by ABCB1 gene, MDR-related protein 1 (MRP1, encoded by ABCC1 gene) and G member 2 of ABC subfamily, also known as breast cancer resistance protein (BRCP), which is encoded by ABCG2 gene (68). The relationship between these three drug transporters and drug resistance in breast cancer has been extensively studied (69–71).

**Figure 4:**
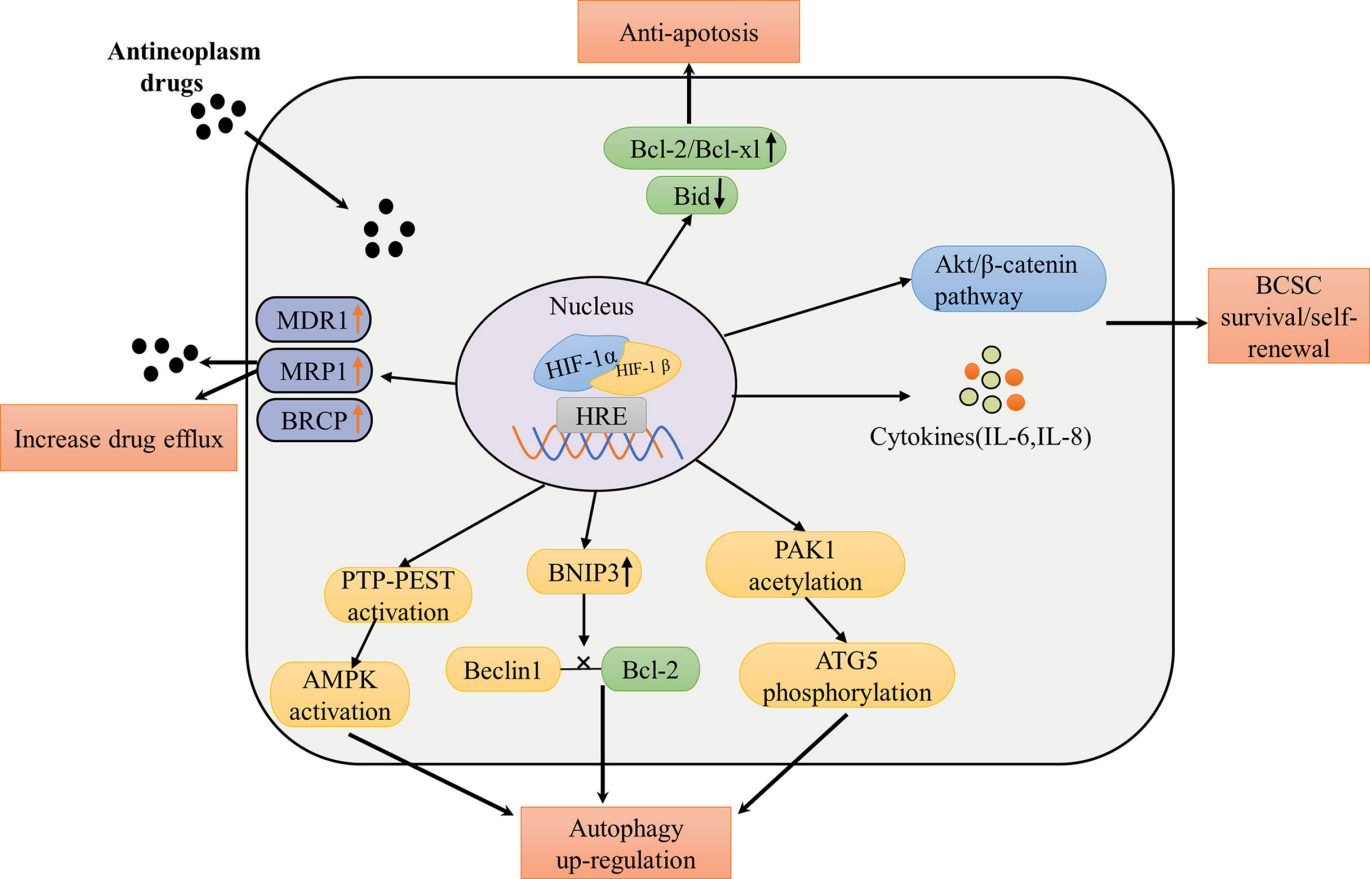
Summary of mechanisms and pathways of HIF-1α mediated drug therapy failure in breast cancer. The pathways of resistance to conventional treatment of HIF-1α include: increasing expression of drug efflux protein leads to drug efllux, increasing expression of anti-apoptotic protein and decreasing expression of pro-apoptotic protein enhance anti-apoptotic effect; phosphorylated Akt/β-catenin pathway and increased cytokine levels promote survival and self-renewal of breast cancer stem cells; promoting the expression of PTP-PEST to activate AMPK, increasing the expression of BNIP3 to interfere the interaction between Beclin1 and Bcl-2 and inducing the acetylation of PAK1to phosphorylate ATG5 promote the upregulation of autophagy. MDR1, multidrug resistance protein 1; MRP1, MDR-related protein 1; BCRP, breast cancer resistance protein; IL, interleukin; ATG5, autophagy-related 5; AMPK, AMP-activated protein kinase; Beclin1, a protein for regulating the formation of autophagosome membranes; Bcl-2 and Bcl-xl, antiapoptotic proteins; Bid, a pro-apoptotic protein; PAK1, p21 activated kinase 1; PTP-PEST, the protein tyrosine phosphatase (PTP)-PEST.

Using quantitative RNA microarray analysis, previous studies revealed an approximately 7-fold increase in MDR in epithelial cells exposed to hypoxia. Meanwhile, MDR1 gene detection identified the binding site between hypoxia-inducible factor-1 (HIF-1) and MDR1. Hypoxia-inducible MDR1expression was significantly inhibited, and the basic MDR1 expression was almost completely lost when HIF-1 expression was inhibited using antisense oligonucleotides (72). Over-expression of the multidrug resistance protein 1 (MDR1, also known as P-glycoprotein or P-gp) is associated with the resistance of taxane and anthracyclines, which are principle chemotherapeutic agents for breast cancer treatment (73). Both the gene encoding MRP1 (ABCC1) and ABCG2 gene encoding BCRP have a hypoxia response element upstream of the open reading frame, and the deletion of this locus prevents hypoxia-dependent activation (74, 75). In one study, western blotting analysis demonstrated increased mRNA and protein expression of MDR1 and MRP1 in SGC7901/HIF cells, whereas hypoxia-induced MDR1 and MRP1 were inhibited in SGC7901/si-HIF cells with HIF-1α knockdown, suggesting that HIF-1α expression can upregulate the expression of drug-resistant proteins MDR1 and MRP1 (76). Another study found that basic HIF-1α protein and BCRP mRNA and protein in AI (letrozole or exemestane)-resistant and HER2-transfected cells were higher than those in AI-sensitive HER2 parents under nonhypoxic conditions and BCRP mRNA in LTLTCa cells (AI-resistance breast cancer cells) treated with CoCl_2_ (HIF-1α stabilizer) increased by about two times compared with the control group. Additionally, in the study, real-time PCR analysis of immunoprecipitated DNA after ChIP found that HIF-1α binds to the hypoxia response element (HRE) region of BCRP promoter in LTLTCa cells under non-hypoxia conditions and CoCl_2_ significantly increased the binding of HIF-1α to BCRP promoter (77). However, the specific signaling pathway utilized by HIF-1α to regulate the expression of drug-resistant proteins remains unclear at present. The level of cell resistance to irinotecan and topotecan was correlated with the expression level of BCRP in cells, which was demonstrated in BCRP-overexpressed breast cancer cells (T47D) (78). These results suggest that HIF-1α expression and stabilization can increase mRNA and protein levels of MDR1, MRP1, and BRCP, which are involved in HIF-1α mediated drug resistance (**Figure 4**).

### HIF-1α mediated BCSC enrichment

Cancer stem cells (CSC) are a kind of cell subpopulation in solid tumors, which possess self-renewal, differentiation, and tumorigenic potential (79). HIF-1α has been reported as a prerequisite for chemotherapy resistance (paclitaxel and gemcitabine) of breast cancer stem cells by inducing ROS-dependent expression of HIF-1α and HIF-2α, leading to HIF-mediated expression of IL-6, IL-8, and MDR1, thereby promoting the survival of BCSCs (80). This study found that exposure of MDA-MB-231, SUM-149, and SUM-159 to paclitaxel increases the percentage of ALDH+ cells that exhibit stem cell properties *in vitro* and *in vivo* by 12-fold. All of the abovementioned effects can be eliminated by the HIF inhibitor digoxin or knockdown of HIF-1α. In addition, an assay of the ALDH activity of MDA-MB-231, SUM159, and MCF-7 cells, which were cultured at 21% O_2_ (normoxia) or 1% O_2_ (hypoxia), demonstrated that the percentage of ALDH+ cells per cell line increased by approximately two to three times and HIF-1α knockdown completely eliminated the hypoxia-induced ALDH+ population increase under hypoxic conditions (81). Furthermore, the authors speculated that HIF-1α promoted stem cell enrichment, in part, through the Akt/β-catenin pathway, which was reported to be a key regulator of CSC self-renewal in breast cancer, because HIF-1α increased levels of both phospho-Akt and phospho-S552 -β-catenin in SUM159 cells, and β-catenin was inactivated (not phosphorylated) when HIF-1α was knocked down (81, 82). Thus, activation of HIF-1α can promote the proliferation and enrichment of tumor stem cells, leading to treatment resistance (**Figure 4**).

### HIF-1α mediated up-regulation of autophagy

Autophagy, also known as cellular self-digestion, is a cellular pathway that involves the degradation of proteins and organelles, with a complex relation to human disease and physiology (83). Autophagy in cancer is a double-edged sword, which can function as a tumor suppressor by preventing the accumulation of damaged proteins and organelles, and as a cell survival mechanism to promote the growth of established tumors under nutritionally deficient or hypoxic conditions (84).

HIF-1α mainly upregulates autophagy in cancer through the following pathways: promoting PTP-PEST expression to activate AMPK, increasing BNIP3 expression, and lastly, interfering with the interaction of Beclin1 with BCL-2, and inducing ELP3-mediated PAK1 acetylation, leading to subsequent PAK1-mediated ATG5 (autophagy-related 5) phosphorylation at T101 residue (**Figure 4**) (85–87). Some studies have revealed that hypoxia increases breast cancer cell resistance to doxorubincin (DOX) with activation of AMPK. Meanwhile, blocking the AMPK-ULK1 pathway can increase the sensitivity of breast cancer (BC) cells to doxorubicin (88, 89). Beclin1, a crucial regulatory protein for regulating autophagosome membrane formation, was upregulated in breast cancer, colorectal cancer, gastric cancer, liver cancer, and cervical cancer and has been associated with chemotherapy resistance (78, 84, 90). The present evidence demonstrated that Beclin1-knockdown breast cancer cells treated with paclitaxel increase cell death by inducing caspase-dependent apoptosis than the group without Beclin1 knockdown (91). In the study, the apoptosis rate of paclitaxel-treated breast cancer cells with Beclin1 knockdown was about 45%, while the apoptosis rate of the group without Beclin1 knockdown was about 33%, and western blot analysis showed that the expression of apoptotic protein caspase-3 increased in the former group. In addition, a study utilizing RT-PCR to measure ATG5 levels in 60 breast cancer tissues found that trastuzumab-resistant patients had higher ATG5 levels than trastuzumab effective patients (92). Similarly, elevated autophagy markers in drug-resistant breast cancer cells have been reported for tamoxifen and fulvestran (93, 94). In summary, it can be concluded that HIF-1α can lead to breast cancer resistance to endocrine drugs and cytotoxic drugs through upregulation of autophagy.

### HIF-1α-mediated inhibition of apoptosis

Apoptosis is a gene-regulated form of cell death that plays a role in biological processes, including embryogenesis, aging, and many diseases (95). Escape from apoptosis is one of the characteristics of cancer cells and is associated with chemotherapy resistance or tumor recurrence (96). At the molecular level, there are two main pathways of apoptosis: external signaling pathways dependent on the binding of death receptor–ligand and internal signaling pathways in response to various cellular stresses (97). Many proteins and cytokines are involved in apoptosis, including members of the B-cell lymphoma-2 (Bcl-2) family, inhibitors of apoptosis-associated proteins, cytochrome c, and the caspase family of proteases (97). Interactions between pro-apoptotic and antiapoptotic members of the Bcl-2 family may mediate the balance between cell survival and apoptosis.

Similar to Bcl-2 family, the role of HIF-1α in apoptosis is also double-sided: promoting apoptosis and inhibiting apoptosis (97). The pro-apoptotic alterations by HIF-1α include down-regulating the expressions of BNIP3, NIX, and NOXA, which belong to the members of the pro-apoptotic Bcl-2 family. In contrast, antiapoptotic effects include increased antiapoptotic proteins such as Bcl-2, Bcl-xL and Myeloid cell leukemia (Mcl-1) and decreased pro-apoptotic Bid, Bax, and Bak levels (98–100). The hypoxia-mediated downregulation of Bid in tumors is reported through HIF-1α dependent mechanisms and contributes to drug resistance (**Figure 4**) (101). HIF-1α was silenced by interfering RNA in HT29 and MEFs cells under hypoxia. At the same time, western blot analysis showed that the Bid protein expression was increased compared with the control group, indicating that the pro-apoptotic protein Bid expression was inhibited when HIF-1α expression was increased under hypoxia, partially explaining the antiapoptotic phenomenon induced by hypoxia. Simultaneously, hypoxia-induced reduction in Bid in HT29 and MEFs cells showed resistance to etoposide. In addition, inhibition of apoptosis induced by overexpression of antiapoptotic proteins is a core factor in acquiring multidrug resistance (MDR) in breast cancer (102). Increased expression of antiapoptotic proteins Bcl-2 and Bcl-xL in HCT116 cells under hypoxia and treatment with irradiation during severe hypoxia significantly improved cell survival scores, which could be ameliorated by Bcl-2 inhibitor ABT-263 (103). This result suggests that hypoxia can increase antiapoptotic proteins and thus resistance to treatment, but the specific mechanism of HIF-1α in this process remains to be explored.

## Targeting HIF-1α directly to overcome drug resistance

Hypoxia-induced overexpression of HIF-1α is an essential factor that induces drug resistance in breast cancer. Therefore, targeting HIF-1α is expected to overcome therapeutic resistance caused by HIF-1α in breast cancer and improve therapeutic efficacy. The drug mechanisms that directly target HIF-1α mainly include inhibiting transcription and translation of HIF-1α and promoting its degradation. Several potential approaches for targeting HIF-1α in breast cancer are described and summarized below (**Table 1**).

**Table 1:** Overview of drugs that inhibit HIF-1 activity reported in breast cancer.

Mechanism	drug name	target	status	Ref
Inhibition of HIF-1α translation	KC7F2	DNA binding	preclinical	(104)
	Digoxin	unknown	approved	(105, 106)
Inhibition of HIF-1α stabilization	AT-533	Hsp90	preclinical	(107)
	STA-9090		clinical trial	(108, 109)
Inhibition of the binding of HIF-1α to the HRE	Echinomycin (NC-13502)	HRE	suspendend	(59)
	liposomal-echinomycin		preclinical	(60)

### Inhibitors of HIF-1α translation

KC7F2, a lead compound with a cysteamine center structure, has been reported to reduce HIF-1α protein levels in a dose-dependent manner (104). Western blotting analysis was performed on LN229 cells incubated with different concentrations of KC7F2 for 6 h under hypoxia conditions and demonstrated that HIF-1α protein levels specifically decreased with the increase of KC7F2 concentration, while β -actin level was basically unaffected. The same results were observed in U251MG, PC3, and MCF-7 cell lines.

Digoxin, a cardiac glycoside, which FDA has identified as a potential inhibitor of HIF-1 activity, has been reported to repress HIF-1α translation. In a study, Hep3B cells were exposed to vector (-) and 100 nM digoxin (+) under hypoxia, followed by a western blot analysis, displaying a significant decrease in HIF-1α protein levels in the digoxin exposed group (105). Another study using digoxin inhibiting HIF-1 from treating breast cancer modeling mice found that the group treated with digoxin had a 78% reduction in tumor growth and a 94% reduction in ipsilateral axillary LN metastasis compared to the control group (106). The mechanism by which cardiac glycosides inhibit HIF-1α may be ROS production leading to HIF-1α ubiquitination and degradation, which is still under investigation.

### Inhibitors of HIF-1α stabilization

HSP90 is a molecular chaperone, and its binding to HIF-1α stabilizes the activity of HIF-1α by blocking VHL-independent proteasome degradation and helping HIF-1α isodimer obtain appropriate conformation to recruit P300 (110). AT-533, a novel Hsp90 inhibitor, is considered a potential candidate for breast cancer treatment, as it inhibits breast cancer growth and angiogenesis by blocking HIF-1α/VEGF/VEGFR-2 signaling pathway (107). Ganetespib (formerly STA-9090) is also a unique Hsp90 inhibitor capable of rapidly inducing degradation of known Hsp90 client proteins (such as HIF-1α) (108, 109). In orthotopic MDA-MB-231 and MDA-MB-435 tumor models, Ganetespib treatment significantly impaired primary tumor growth and inhibited local tumor invasion and distant tumor metastasis to regional lymph nodes and lungs (111).

### Inhibition of the binding of HIF-1α to the HRE

HRE is the DNA binding site of HIF-1α, which promotes the expression of HIF-1α-related target genes. Echinomycin (NC-13502) has a strong hypoxic selective cytotoxicity by inhibiting the binding of HIF to VEGF promoter HRE but does not affect HIF to AP-1 or NF-κB promoter HRE (59). Chromatin immunoprecipitation studies have shown that echinomycin can also inhibit HIF-1 binding to DNA. It was reported that the liposomal -echinomycin can effectively inhibit HIF-1α transcriptional activity of primary and metastatic TNBC cells and inhibit tumor growth *in vivo* (60). In the study, liposome-echinomycin treatment in xenograft mice (MDA-MB-231 and SUM-159) significantly inhibited tumor volume and almost eradicated liver and lung metastases in both models compared with the control group.

## The limitations of clinical application of HIF-1α inhibitors

Although these compounds targeting HIF-1α have shown efficacy *in vitro*, HIF-1α inhibitors still have several limitations. First, differential expression of HIF-1α limits the efficacy of anti-HIF-1α therapy. Second, HIF-1α inhibitors monotherapy have limited efficacy (61). In a phase II clinical trial, 2ME2 NCD showed no efficacy in patients with renal cell carcinoma (61). Similarly, in another Phase II trial, 17-AAG (tanespimycin), a potential HSP90 inhibitor that increased HIF-1α degradation, did not achieve objective response rates in the treatment of metastatic RCC (62). Because no clinical trials investigated HIF-1α inhibitors monotherapy in breast cancer yet, the efficacy of HIF-1α inhibitors monotherapy in breast cancer may not satisfactory either. Finally, HIF-1α was measured by western blotting, real-time quantitative PCR (RT-PCR), and immunostaining. These detection methods are mainly used in cancer research. The clinical implementation requires invasive tissue biopsy which may not be feasible to patients with late-stage breast cancer. Therefore, non-invasive tests for HIF-1α are highly desired.

Given the limitations of HIF-1α inhibitors, the combination of HIF-1α inhibitors with chemotherapeutic agents or other agents may achieve an optimal efficacy. Because the relevant combination therapies in breast cancer still stay in the pre-clinical stage, we are unable to draw a solid conclusion yet. Nevertheless, a previous preclinical study reported that digoxin increased the sensitivity of triple negative breast cancer to paclitaxel and gemcitabine *in vivo* (80). Acriflavine, a HIF-1α dimerization inhibitor, has also been reported to enhance the antitumor activity of sunitinib in 4T1 breast cancer models (112). Additional data from *in vitro* and *in vivo* studies are urgently needed to determine whether the use of HIF-1α inhibitors in combination with current therapies may be beneficial for breast cancer patients. There is also an urgent need for combinations of HIF-1α inhibitors to be tested in clinical trials, especially in patients with drug resistance.

## Conclusion

Since the HIF family transcription factors were first discovered nearly 30 years ago, great progress has been made in understanding their regulation and role in physiology and pathophysiology. This achievement ultimately led to the awarding of the 2019 Nobel Prize in Physiology or Medicine for the discovery of HIF, the clinical approval of multiple therapies affecting upstream and downstream targets of the HIF subtype, and the clinical development of first-in-class selective inhibitors of HIF-2α. HIF subtypes may play complementary roles in driving tumor progression due to their nonoverlapping spatiotemporal regulation in tumor cells and tumor microenvironment (TME) cells. In brief, HIF-1α promotes metabolic reprogramming in tumor cells and TME cells, while HIF-2α induces an aggressive stem-like phenotype within tumor cells, and both contribute to angiogenesis and the production of tumor-licensed TME.

Tumor cell resistance to therapeutic drugs is a thorny problem, limiting the success in the clinical treatment of breast cancer. HIF-1α is upregulated in different breast cancer subtypes and is associated with poor prognosis and drug resistance in breast cancer. HIF-1α confers resistance to conventional therapies through several signaling pathways involved in BCSC enrichment, drug outflow, apoptosis, and autophagy. At present, many compounds can directly or indirectly inhibit HIF-1α to alleviate drug resistance, but most of them are limited by poor efficacy or large toxic side effects *in vivo*, which may present challenges in the future. Due to the inherent limitations of cellular and animal models, a deep understanding of the role of HIF transcription factors in TME in clinical setting is critical to understanding the determinants of therapeutic resistance and to develop relevant compounds targeting them. Given these problems, we can consider the optimization method from the following three points. First, in addition to HIF-1α, HIF-2α has also been associated with histological grade, Ki67 expression, and multidrug resistance in breast cancer (113). Therefore, further exploration of HIF-2α may find another effective drug target. Second, HIF-1α varies between and within breast cancer subtypes and considering breast cancer patient selection may help screen for effective drugs. Finally, it can be considered that delivering drugs with nanoscale liposomes may reduce side effects and achieve both efficacy and safety. Most drugs remain in development, and some are in clinical trials, so combinations of drugs targeting HIF-1α and other drugs are unavailable. No single therapy can completely solve the problem of breast cancer drug resistance, so combination therapy is the best option in the future. Because inhibition of one HIF-α subtype tends to induce expression of the remaining subtypes, we propose that, in some cases, direct targeting of these two HIF subtypes may provide more benefits than targeting each subtype alone.

## Author contributions

FC and YW designed the conceptualization; LY and ST wrote the manuscript. HY, HZ, and YZ made manuscript review and critical comments. All authors contributed to the article and approved the submitted version.

## Funding

This article was supported by the Shanghai Yangpu District Health and Family Planning Commission Fund for Hao Yi Shi Training Project (Grant no. 202056, 2020-2023) and the Natural Science Foundation of Shanghai (Grant no. 18ZR1436000).

## Acknowledgments

We thank Home for Researchers (www.home-for-researchers.com) for language polishing.

## Conflict of interest

The authors declare that the research was conducted in the absence of any commercial or financial relationships that could be construed as a potential conflict of interest.

## Publisher’s note

All claims expressed in this article are solely those of the authors and do not necessarily represent those of their affiliated organizations, or those of the publisher, the editors and the reviewers. Any product that may be evaluated in this article, or claim that may be made by its manufacturer, is not guaranteed or endorsed by the publisher.
